# LAG3-PD-1 Combo Overcome the Disadvantage of Drug Resistance

**DOI:** 10.3389/fonc.2022.831407

**Published:** 2022-04-14

**Authors:** Yiming Wei, Zhaoming Li

**Affiliations:** Department of Oncology, The First Affiliated Hospital of Zhengzhou University, Zhengzhou, China

**Keywords:** Lymphocyte Activation Gene-3 (LAG-3), immune checkpoint, drug resistance, Programmed Cell Death 1 (PD-1), immunotherapy

## Abstract

Although PD-1 blockade therapy has been promising in cancer treatment, only 4% (pancreatic cancer) to 70% (melanoma) of patients have a positive response to this blockade therapy, which is one of its important disadvantages. Therefore, it is important to seek out new targets for cancer immunotherapy to improve the overall response rate in patients. Lymphocyte activation gene-3 (LAG-3), an immune checkpoint receptor, is mainly expressed in activated immune cells. LAG-3 maintains the body’s immune homeostasis under physiological conditions while mediating tumour immune escape. Several preclinical and clinical examinations have shown that LAG-3 blockade effectively alleviates the patient’s tolerance to PD-1 immune checkpoint inhibitors. Moreover, the combination of LAG-3 and PD-1 blockade has good clinical efficacy in cancers. Hence, synchronous LAG-3 and PD-1 inhibition may be a potential new strategy for tumour immunotherapy.

## Introduction

Tumour cells can evade the recognition and killing of the immune system of the body with the help of immune checkpoint receptors (1). Thus, blocking immune checkpoint receptors might be a widely effective method of tumor immunotherapy. Currently, although anti-PD-1/PD-L1 antibodies (2) are relatively mature, similar to anti-CTLA4 antibodies, the overall response rate is low in patients because of drug resistance (3, 4). Therefore, finding new tumour immunotherapy targets is urgent. As a new immune checkpoint receptor, LAG-3 plays a vital role in immune homeostasis maintenance and tumour immune escape and is widely present in various activated immune cells (5, 6). The combination of PD-1 and LAG-3 blockade may be a new treatment for drug-resistant patients.

## LAG-3 Expression and Ligand

As a type I transmembrane protein, the LAG-3 molecule contains three domains: a transmembrane domain, an extracellular domain, and an intracellular domain. Four immunoglobulin superfamily domains constitute the extracellular domain of the LAG-3 molecule, which binds to its ligand. There are three parts of the transmembrane domain and intracellular domain: the highly conserved KIEELE motif (7), the potential serine phosphorylation site S454, and the glutamate-proline-rich sequence. The glutamate-proline rich sequence is also known as the EP repeated sequence and is closely related to the signal transduction of the intracellular region (8).

LAG-3 is expressed widely on different cells: (i) T-cell subpopulations including activated CD4+ T helper cells (Th) and cytotoxic CD8+ T cells (CTL) (9–12). T-cell activation is a necessary condition for LAG-3 expression on T cell subpopulations (13). The LAG-3 level is closely associated with the expression levels of IL-2, IL-7, and IL-12 (14). Additionally, activated CD4+ effector T cells have been shown to express LAG-3, especially on activated natural regulatory T cells (nTregs) and inducible T-regulatory cells (iTregs) (15). (ii) Natural killer (NK) cells and invariant NKT cells (16). (iii) LAG-3+CD138hi plasma cells or regulatory B cells (Bregs), which suppress the immune system through upregulation of IL-10 expression (17). (iv) Other cells, such as plasmacytoid dendritic cells (pDC) and neuronal cells, which do not belong to the lymphocyte lineage (18).

As a typical ligand of LAG-3, MHC-II interacts with LAG-3 through the domain of D1 (19). LAG-3 has a higher affinity for MHC-II than CD4 molecules (20). Significantly, LAG-3 may be cleaved by metalloproteases and release a soluble form of LAG-3(sLAG-3) (21). It remains unclear whether sLAG-3 has the same high affinity for MHC-II as LAG-3. A previous study showed that MHC-II signalling induced specifically by sLAG-3 severely impaired the differentiation of monocytes (22). In contrast, another study showed that naturally cleaved sLAG-3 does not specifically bind with MHC-II, and only the cell surface LAG-3 dimer or the dimeric LAG-3:Ig fusion protein possesses a high affinity for MHC-II (21). The function and mechanism of sLAG-3 remain to be further explored. LAG-3 negatively modulates T-cell activation and the production of related cytokines by transmitting blocking signals *via* its cytoplasmic domain (23). Studies have shown that the combination of LAG-3 and MHC-II contributes to avoiding apoptosis of tumour cells and promoting tumour-specific CD4+ T-cell recruitment but reduces the response of CD8+ T cells (7, 13, 23–27). As the second ligand of LAG-3, galectin-3 (Gal-3) shows high expression in multiple cancer cells and activated T lymphocytes and modulates T-cell activation. The cell toxicity of CD8+ T cells can be inhibited by binding to LAG-3 (28, 29). Additionally, fibrinogen-like protein 1 (FGL1), produced by the liver, is a recently identified ligand of LAG-3 (30). As one member of the fibrinogen family, FGL1 muffles antigen-specific T cells by binding to LAG-3 (31). The expression of FGL1 is related to IL-6 *via* JAK2/STAT3 signalling (32). Studies have shown that FGL1 is abundantly secreted in multiple cancers, including lung cancer, prostate cancer, melanoma, and colorectal cancer (33).

## The Immunosuppressive Function of LAG-3

The interplay between MHC-II and LAG-3 can lower the growth capacity of CD4+ T cells and the secretion of cytokines (13). Moreover, the addition of LAG-3 antibodies can reinstate the activity of CD4+ T cells (34). LAG-3 does not bind to all MHC-II molecules but selectively recognizes and combines antigen peptide-MHC-II complexes (pMHC-II) and controls the pMHC-II-mediated CD4+ T-cell response (35). It has been proven that LAG-3 is an independent negative regulator and that there is no competitive relationship with other regulatory molecules, such as CD4 (35). Experiments have shown that LAG-3 restrains CD4+ T- cell functions by directly transmitting inhibitory signals through the intracellular region but does not block the interaction between CD4-MHC-II or TCR-MHC-II (8, 23, 35).

The activity of CD8+ T cells was higher in LAG-3 knockout (KO) mice than in wild-type mice. Low-level LAG-3 expression was noted in the initial activation of CD8+ T cells, and LAG-3 expression increased after tumour antigen stimulation. Additionally, LAG-3 could significantly inhibit the cytotoxicity of CD8+ T cells (36). In addition, a previous study showed that LAG-3 directly suppressed CD8+ T cells through signal transduction but did not rely on the interaction between CD4+ T cells and MHC-II (35).

Treg cells, including nTreg and iTreg cells, negatively regulate immunity and can decrease the activity of T cells. LAG-3 was capable of inducing the activation of Treg cells and activating their immunosuppressive function (15, 37). LAG-3 is expressed on both activated natural Tregs (nTregs) and induced CD4+ FoxP3+ Treg (iTreg) cells (15). The addition of LAG-3 antibodies can significantly control the activity of Treg cells. Compared with wild-type mice, the negative regulatory function of nTreg cells was significantly downregulated in LAG-3 KO mice (15, 38, 39).

**Figure 1:**
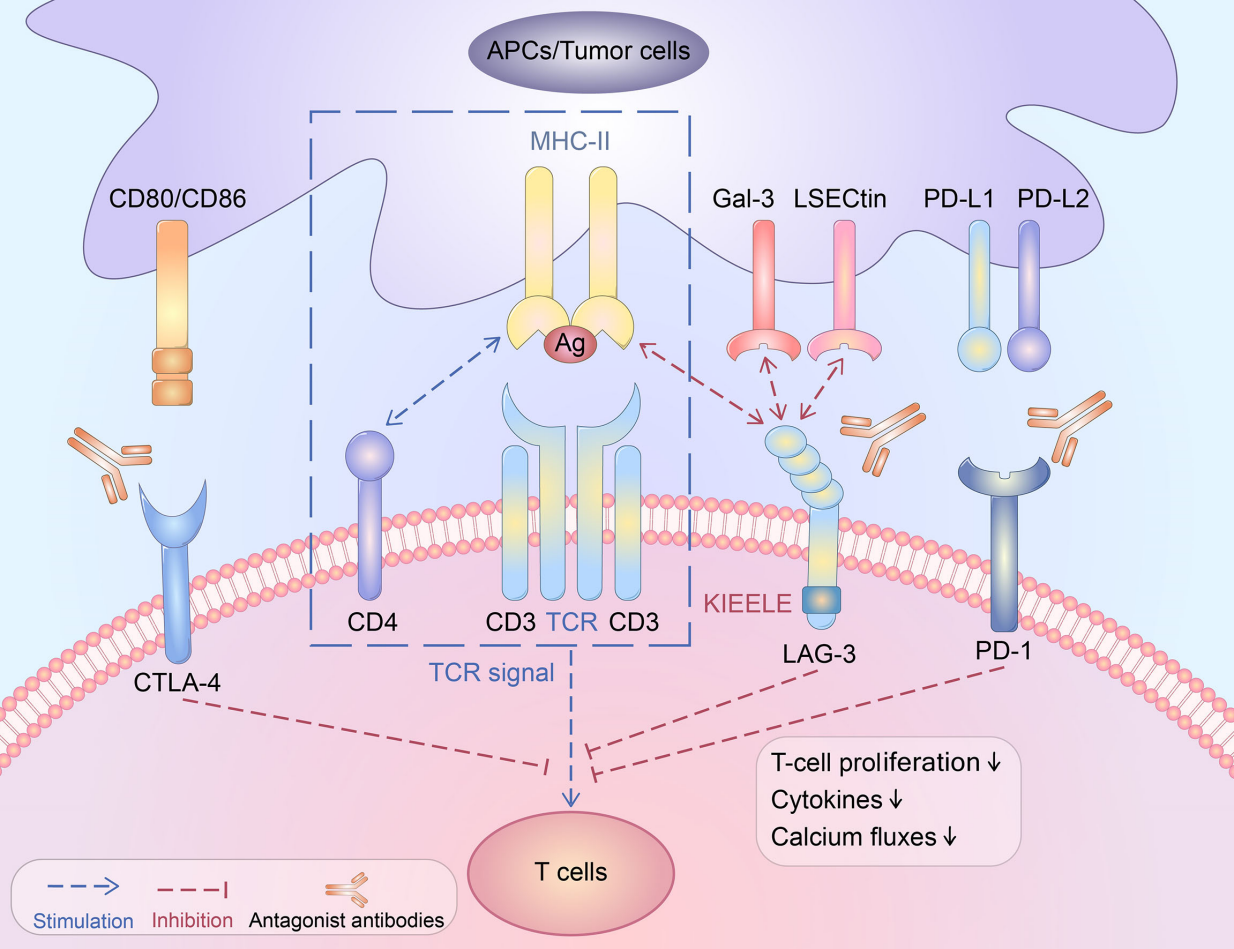
The immunosuppressive function of LAG-3.

Although the LAG-3 inhibition-induced signalling pathway remains unclear, several studies have discussed this issue. Experiments have shown that LAG-3 is highly correlated with CD3 and that the cross-linking of these two molecules can block the proliferation of T cells, cytokine production, and calcium production (**Figure 1**). LAG-3 may downregulate the immune response by interfering with TCR signal-transduction (40). Furthermore, the inhibition of LAG-3 in effector CD4+ T cells occurs in a KIEELE motif-dependent manner (23). LAG-3 may transduce two independent inhibitory signals through the KIEELE motif and the FSAL motif in the EP repeat sequence. Both the FSAL motif and KIEELE motif are key points in LAG-3-induced signalling pathways (8). However, how these motifs regulate the TCR response and downstream molecules is still unclear.

## LAG-3 and Tumour Immunity

MHC-II and FGL-1, as ligands of LAG-3, are related to LAG-3-mediated tumour immune escape (19, 20, 41). MHC-II recruits CD4+ T cells and enhances antitumour immunity in the early stage of tumorigenesis. The activity and expression level of the LAG-3 molecule increases on the surface of TIL cells with the development of tumours. After binding to MHC-II, LAG-3-induced antitumour immunity turns into immunosuppression. For example, MHC-II-expressing melanoma cells block the functions of tumour-infiltrating CD4+ T cells, thus evading the recognition and killing of the immune system (42). FGL-1 is secreted by the liver (30). Under normal circumstances, the expression of FGL-1 is low in hepatocytes, and its expression markedly increases when cancers occur (31, 33).

Generally, the LAG-3 molecule detected on T cells is regarded as a marker of aggressive progression of cancers. Under the stimulation of tumour antigens, lymphocytes highly express LAG-3 (43, 44). Furthermore, the LAG-3 expression level is obviously related to the survival rate and prognosis of inpatients (45). For example, the number of CTLA-4+LAG-3+ T-cell subsets significantly increased in AML patients, and the production level of LAG-3 was closely related to the classification of patients with AML (46). LAG-3 presented increased expression on the TILs of NSCLC patients (47, 48), and high LAG-3 was also found in nonadenocarcinoma tissues (47). LAG-3 expression in T cells from the peripheral blood of soft tissue sarcoma patients was higher than that in healthy people. LAG-3 is mainly produced and localizes to CD8+ TILs in individuals with soft tissue sarcoma. High LAG-3 expression is related to late-stage disease, high pathological grade, and low survival rates (49). The phenomenon is most obvious in CD8+ TILs in which LAG-3 is highly expressed and combined with its ligand and is detected on the cancer cell surface, leading to the decline or exhaustion of T-cell function and thereby promoting tumour immune escape (36). For example, the overexpression of LAG-3 was detected on CD8+ TILs in ovarian cancer tumour tissues, and cytokine secretion was strongly decreased (50, 51). Although the number of endogenous CD8+ T cells was increased in Hodgkin’s lymphoma patients, their response was low. Moreover, their function was negatively associated with LAG-3 expression on TILs (52–54). The gene expression profile data showed that LAG-3 caused T-cell exhaustion in the tumour microenvironment by cooperating with a variety of inhibitory receptors in patients with melanoma (6, 55, 56).

Chemotherapy affected the expression levels of LAG-3 and PD-1, which ultimately mediated tumour immune escape. The proportion of CD8+LAG3+PD-1+ T cells was significantly higher in patients receiving preoperative paclitaxel plus platinum chemotherapy than in patients receiving surgery alone (57).

## Combined Anti-LAG-3 and Anti-PD-1 Blocking

Immune homeostasis is maintained by the balance between costimulatory and inhibitory signals. Increased checkpoint receptors can alleviate antigen-specific T-cell activation, bringing about a proinflammatory response lower than normal circumstances (6). After long-term activation of tumour antigens, checkpoint receptor expression is maintained, which causes effector T cells to enter an “exhaustion” state. Exhausted T cells show a gradual decrease in proliferation capacity and loss of function, including the production of inflammatory cytokines and degranulation (58, 59). Multiple clinical trials have shown that blocking the immune checkpoint PD-1 can significantly improve the clinical treatment effect in malignant tumours by restoring the function of effector T cells. Unfortunately, only a few patients benefit from this therapy because of the development of drug resistance mechanisms within the tumour microenvironment (60–63). The latest data showed that LAG-3 might also be vital in the development of resistance to the curing of PD-1 or PD-L1 by inhibiting the activity and proliferation of CD8+ T cells and increasing the inhibitory activity of Tregs (64–66). LAG-3 can regulate the activity of PD-1+ cells (67). LAG-3 and PD-1 synergistically regulate T-cell function. Combined anti-LAG-3 and anti-PD-1 antibody treatment has shown a strong antitumour effect in mice resistant to single-antibody treatment. Excitingly, there was no obvious evidence of autoimmunity, thus suggesting the possibility of clinical efficacy and safety by combining anti-LAG-3 and anti-PD-1 antibody treatment (36).

Currently, several molecules targeting LAG-3 are in clinical development (**Table 1**). Although these molecules were well tolerated, the effect of single-molecule-based therapy was limited. Some animal experiments have implied that LAG-3 might be a potential target in combination with anti-PD-1. (i) Both LAG-3 and PD-1 were observed on T cells in the tumour microenvironment in animal models of mouse MC38 colorectal adenocarcinoma and SalN fibroma. Furthermore, the antitumour efficacy of combined immunotherapy far exceeded that of any single immunotherapy (36). (ii) In a mouse model of ovarian cancer, tumour-infiltrating T cells coexpressed LAG-3 and PD-1. Moreover, LAG-3 and PD-1 blockade upregulated effector T-cell activity, thereby inhibiting tumour growth (51). (iii) Both LAG-3 and PD-1 blockade caused IFN-γ secretion and CD8+ T-cell cytotoxicity upregulation in melanoma (68). (iv) Another study on mouse prostate cancer showed that compared with inhibition of a single target, the combined suppression of PD-1 and LAG-3 markedly improved the antitumour effect of antitumour vaccines (69). (v) Recently, a new fully human anti-LAG-3 therapeutic IgG4 antibody was developed (REGN3767). Furthermore, the combination of REGN3767 and PD-1 antibody showed higher antitumour efficacy and accelerated the production of proinflammatory cytokines in tumour-specific T cells from mouse tumour models established using humanized PD-1xLAG-3 mice. REGN3767 had good pharmacokinetics and toxicology in nonhuman primates, showing good clinical application prospects (70). (vi) In a humanized mouse non-small cell lung cancer model, the LAG-3 antibody TSR-033 enhanced the efficacy of anti-PD-1 monotherapy and increased immune system activation (71). (vii) FS118, a bispecific antibody against PD-L1 and LAG-3, has been confirmed to enhance the activation of T cells in mouse tumour models, bringing about potent antitumour activity (72).

**Table 1:** Main Ongoing Clinical Trials of anti- LAG-3 Combined with anti-PD-1 antibody.

NO.	NCT Number	Title	Tumor	Interventions	Phases
1	NCT02658981	A Phase I Trial of Anti-LAG-3 or Anti-CD137 Alone and in Combination With Anti-PD-1 in Patients With Recurrent GBM	GBM	LAG-3 Antibody; PD-1 Antibody	Phase 1
2	NCT03250832	A Phase 1 Dose Escalation and Cohort Expansion Study of TSR-033, an Anti-LAG-3 Monoclonal Antibody, Alone and in Combination With an Anti-PD-1 in Patients With Advanced Solid Tumors	Solid Tumors	TSR-033;Dostarlimab;mFOLFOX6;FOLFIRI;Bevacizumab	Phase 1
3	NCT04080804	Study of Safety and Tolerability of Nivolumab Treatment Alone or in Combination With Relatlimab or Ipilimumab in Head and Neck Cancer	HNSCC	Nivolumab;Relatlimab;Ipilimumab	Phase 2
4	NCT04140500	Dose Escalation Study of a PD1-LAG3 Bispecific Antibody in Patients With Advanced and/or Metastatic Solid Tumors	Solid Tumors	RO7247669	Phase 1
5	NCT01968109	An Investigational Immuno-therapy Study to Assess the Safety, Tolerability and Effectiveness of Anti-LAG-3 With and Without Anti-PD-1 in the Treatment of Solid Tumors	Solid Tumors	Relatlimab;Nivolumab;BMS-986213	Phase 1/2
6	NCT04370704	Study of Combination Therapy With INCMGA00012 (Anti-PD-1), INCAGN02385 (Anti-LAG-3), and INCAGN02390 (Anti-TIM-3) in Participants With Select Advanced Malignancies	Melanoma	INCAGN02385;INCAGN02390;INCMGA00012	Phase 1/2
7	NCT02061761	Safety Study of Anti-LAG-3 in Relapsed or Refractory Hematologic Malignancies	HematologicNeoplasms	BMS-986016;BMS-936558	Phase 1/2
8	NCT03005782	Study of REGN3767 (Anti-LAG-3) With or Without REGN2810 (Anti-PD1) in Advanced Cancers	Solid Tumors	REGN3767;cemiplimab	Phase 1
9	NCT03311412	Sym021 Monotherapy, in Combination With Sym022 or Sym023, and in Combination With Both Sym022 and Sym023 in Patients With Advanced Solid Tumor Malignancies or Lymphomas	Tumor	Sym021;Sym022;Sym023	Phase 1
10	NCT04641871	Sym021 in Combination With Either Sym022 or Sym023 in Patients With Advanced Solid Tumor Malignancies	Solid Tumors	Sym021;Sym022;Sym023	Phase 1
11	NCT03743766	Nivolumab, BMS-936558 in Combination With Relatlimab, BMS-986016 in Patients With Metastatic Melanoma Naïve to Prior Immunotherapy in the Metastatic Setting	Melanoma	Relatlimab;Nivolumab;	Phase 2
12	NCT04618393	A Study of EMB-02 in Participants With Advanced Solid Tumors	Solid Tumors	EMB-02	Phase 1/2
13	NCT04785820	A Study of RO7121661 and RO7247669 Compared With Nivolumab in Participants With Advanced or Metastatic Squamous Cell Carcinoma of the Esophagus	Esophageal Squamous Cell Carcinoma	RO7121661;RO7247669;Nivolumab	Phase 2
14	NCT03219268	A Study of MGD013 in Patients With Unresectable or Metastatic Neoplasms	Solid Tumors	MGD013;margetuximab	Phase 1
15	NCT03623854	Nivolumab and Relatlimab in Treating Participants With Advanced Chordoma	Chordoma	Nivolumab;Relatlimab	Phase 2
16	NCT04634825	Enoblituzumab Plus Retifanlimab or Tebotelimab in Head and Neck Cancer	HNSCC	Enoblituzumab;Retifanlimab;Tebotelimab	Phase 2
17	NCT02966548	Safety Study of BMS-986016 With or Without Nivolumab in Patients With Advanced Solid Tumors	Solid Tumors	Relatlimab;Nivolumab	Phase 1
18	NCT03459222	An Investigational Study of Immunotherapy Combinations in Participants With Solid Cancers That Are Advanced or Have Spread	Solid Tumors	Relatlimab;Nivolumab;BMS-986205;Ipilimumab	Phase 1/2
19	NCT03044613	Nivolumab +/- Relatlimab Prior to Chemoradiation With II/III Gastro/Esophageal Cancer	Esophageal Cancer	Nivolumab;Relatlimab;Carboplatin;Paclitaxel;Radiation: Radiation	Phase 1
20	NCT04326257	Personalized Immunotherapy in Patients With Recurrent /Metastatic SCCHN That Have Progressed on Prior Immunotherapy	HNSCC	Nivolumab+Relatlimab;Nivolumab+Ipilimumab	Phase 2
21	NCT04913922	Relatlimab With Nivolumab and 5-Azacytidine for the Treatment of AML	Acute Myeloid Leukemia	Azacitidine Injection;Nivolumab;Relatlimab	Phase 2
22	NCT03365791	PDR001 Plus LAG525 for Patients With Advanced Solid and Hematologic Malignancies	Solid and Hematologic Malignancies	PDR001;LAG525	Phase 2
23	NCT03642067	Study of Nivolumab and Relatlimab in Patients With Microsatellite Stable (MSS) Advanced Colorectal Cancer	Colorectal Adenocarcinoma	Nivolumab;Relatlimab	Phase 2
24	NCT03607890	Study of Nivolumab and Relatlimab in Advanced Mismatch Repair Deficient Cancers Resistant to Prior PD-(L)1 Inhibitor	Solid Tumors	Nivolumab;Relatlimab	Phase 2
25	NCT04658147	Feasibility and Efficacy of Perioperative Nivolumab With or Without Relatlimab for Patients With Potentially Resectable Hepatocellular Carcinoma (HCC)	Hepatocellular Carcinoma	Nivolumab;Relatlimab	Phase 1

Abbreviations: Relatlimab, anti-LAG3 antibody; RO7247669, an anti PD-1 and LAG-3 bispecific antibody; BMS-986016, anti-LAG-3; BMS-936558, anti-PD-1 Monoclonal Antibody (Nivolumab); EMB-02, a Bi-specific Antibody Against PD-1 and LAG-3; RO7121661, a PD1-TIM3 Bispecific Antibody; RO7247669, a PD1-LAG3 Bispecific Antibody; MGD013, A Bispecific DART® Protein Binding PD-1 and LAG-3; PDR001, PD-1 IgG4 antibody; LAG525, LAG-3 antibody; HNSCC, Head and Neck Squamous Cell Carcinoma; GBM, Glioblastoma.

As an IgG4κ bispecific DART^®^ molecule that combines LAG-3 and PD-1, tebotelimab could disrupt nonredundant blocking pathways and further restore the function of exhausted T cells. In an open-label, randomized, phase II/III MAHOGANY trial (NCT04082364), tebotelimab reversed PD-1- and LAG-3-mediated inhibitory effects by controlling the interaction with PD-L1/PD-L2 or MHC-II molecules, thereby restoring exhausted T-cell function and enhancing antitumour immunity. Additionally, dual inhibition of PD-1 and LAG-3 improved the effectiveness of HER2 antibodies by increasing the innate and adaptive immune response against HER2-overexpressing cancer cells (73).

Eftilagimod alpha (efti, IMP321 or LAG-3Ig), a soluble LAG-3 protein and MHC-II agonist, activates APC, causing CD8+ T-cell activation. An open-label, multicentre, dose-escalation study in phase I advanced melanoma invalids was performed to assess the safety, tolerability, pharmacokinetics, and pharmacodynamics of the combined efti and PD-1 antibody. Twenty-four melanoma patients received pembrolizumab and subcutaneous efti injections at doses of one mg, six mg, or thirty mg biweekly. The main adverse event of efti was a reaction at the injection site. Dose-limiting toxicity was not reported. The count of activated CD8+ T cells and CD4+ T cells and the IFN-γ expression levels were increased after subcutaneous injection of efti. More importantly, a 33% overall response rate (ORR) was still observed in some patients who were resistant to pembrolizumab. The clinical trial showed that the combination of efti and pembrolizumab is well tolerated and has encouraging antitumour activity (68).

## Conclusion and Outlook

Although PD-1 blockade has undergone a paradigm shift in multiple malignant cancers, most tumours show a high rate of primary resistance to this drug. Multiple preclinical and clinical data showed that the double-checkpoint inhibition of LAG-3 and PD-1 can be an application for overcoming ICB resistance. Therefore, combined PD-1 and LAG-3 inhibition may be a promising immunotherapy program for cancers. However, the synergistic effects based on anti-PD-1 and anti-LAG-3 need to be further confirmed in different tumours through more clinical trial data.

## Author Contributions

YW and ZL contributed to the conception of the study and wrote the manuscript. All authors contributed to the article and approved the submitted version.

## Funding

Education Department of Henan Province, Key Scientific Research Project plan of Colleges and Universities in Henan Province, Project No.21A320027.

## Conflict of Interest

The authors declare that the research was conducted in the absence of any commercial or financial relationships that could be construed as a potential conflict of interest.

## Publisher’s Note

All claims expressed in this article are solely those of the authors and do not necessarily represent those of their affiliated organizations, or those of the publisher, the editors and the reviewers. Any product that may be evaluated in this article, or claim that may be made by its manufacturer, is not guaranteed or endorsed by the publisher.
